# Eu^3+^-activated La_2_MoO_6_-La_2_WO_6_ red-emitting phosphors with ultrabroad excitation band for white light-emitting diodes

**DOI:** 10.1038/s41598-017-12161-5

**Published:** 2017-09-20

**Authors:** Peng Du, Jae Su Yu

**Affiliations:** Department of Electronic Engineering, Kyung Hee University, Yongin-si, 446-701 Republic of Korea

## Abstract

A series of novel Eu^3+^-activated La_2_MoO_6_-La_2_WO_6_ red-emitting phosphors have been successfully prepared by a citrate-assisted sol-gel process. Both photoluminescence excitation and emission spectra suggest that the resultant products have the strong ultrabroad absorption band ranging from 220 to 450 nm. Under the excitation of 379 nm, the characteristic emissions of Eu^3+^ ions corresponding to the ^5^D_0_ → ^7^F_*J*_ transitions are observed in the doped samples. The optimal doping concentration for Eu^3+^ ions is found to be 12 mol% and the quenching mechanism is attributed to the dipole-dipole interaction. A theoretical calculation based on the Judd-Ofelt theory is carried out to explore the local structure environment around the Eu^3+^ ions. The studied samples exhibit a typical thermal quenching effect with a T_0.5_ value of 338 K and the activation energy is determined to be 0.427 eV. A near-ultraviolet (NUV)-based white light-emitting diode (LED) is packaged by integrating a mixture of resultant phosphors, commercial blue-emitting and green-emitting phosphors into an NUV LED chip. The fabricated LED device emits glaring white light with high color rendering index (84.6) and proper correlated color temperature (6492 K). These results demonstrate that the Eu^3+^-activated La_2_MoO_6_-La_2_WO_6_ compounds are a promising candidate for indoor lighting as red-emitting phosphors.

## Introduction

By virtue of admirable advantages of long working lifetime, energy saving, low cost, high luminous efficiency and environmental compatibility, the phosphor-converted white light-emitting diodes (WLEDs) which are considered as the next-generation illumination sources to supersede the conventional fluorescent lamps have been extensively used in indoor lighting, automobile displays and flashlights^1–5^. Presently, the commercial WLEDs which are made up of a blue-emitting InGaN LED chip and Y_3_Al_5_O_12_:Ce^3+^ yellow-emitting phosphors suffer from poor color rending index (CRI ~ 70–80) and high correlated color temperature (CCT ~ 7000 K) as a result of inefficient red emission component^6–8^. To circumvent these drawbacks, a new strategy utilizing the near-ultraviolet (NUV) LED chip to pump the hybrid tricolor (blue, green and red) phosphors is performed to emit warm white light^9–11^. From the aforementioned combinations, one knows that the eventual behaviors of WLED devices can be significantly affected by the phosphors and they are expected to be efficiently excited by NUV or blue light. In comparison with commercial blue-emitting and green-emitting phosphors, the current red-emitting phosphors, by taking Y_2_O_2_S:Eu^3+^ for example, still exhibit some unsatisfied characteristics such as weak absorption in the NUV/blue region, low luminous efficiency and poor stability^12,13^. In order to improve the performance of red-emitting phosphors, the nitride- and germanide-based red-emitting phosphors, such as Ca_2_Si_5_N_8_:Eu^2+^, Sr[LiAl_3_N_4_]:Eu^2+^, Sr_3_Y_2_Ge_3_O_12_:Eu^2+^ and Sr_2_GeO_4_:Eu^2+^, were developed^14–17^. Unfortunately, to synthesis these compounds, a reduced atmosphere and a high sintering temperature are required, leading to high investment as well as environmental issues. Therefore, the development of novel red-emitting phosphors that can be excited by NUV or blue light is highly desirable.

Nowadays, tremendous interests have been attracted in rare-earth (RE) ions-based luminescent materials because of their potential feasibility in many fields of solar cells, thermometry, field emission displays, WLEDs and biomedicine^18–22^. In comparison, the Eu^3+^ ion, as an obbligato member of RE ions, is most frequently used as a red-emitting activator since its narrow red emission originating from ^5^D_0_ → ^7^F_2_ transition^23,24^. Up to date, some Eu^3+^-activated red-emitting phosphors, such as Li_3_Ba_2_Y_3_(WO_4_)_8_:Eu^3+^, Ca_2_Ga_2_SiO_7_:Eu^3+^ and Y_2_Mo_4_O_15_:Eu^3+^, were successfully synthesized^25–27^. However, these red-emitting phosphors suffer from narrow excitation bands and do not match well with the emitting band of NUV or blue LED chip which limits their promising applications in WLEDs. To solve this problem, an appropriate luminescent host material should be selected. According to previous literatures, one obtains that the tungstates and molybdates are promising candidates for luminescent host materials because of their outstanding metrics of high stability, low phonon energy, relatively low synthetic temperature and admirable intrinsic luminescent performance^28–30^. Meanwhile, both the tungstates and molybdates exhibit a broad absorption band in the UV region arising from the charge transfer (CT) transitions of O^2−^ → W^6+^ and O^2−^ → Mo^6+^, respectively^31,32^. Furthermore, owing to the small difference in the ionic radii between W^6+^ and Mo^6+^ ions, they can be easily substituted by each other, leading to the formation of molybdates-tungstates compounds^12,32^. It was revealed that the absorption bands of the Eu^3+^-activated tungstates-molybdates compounds were shifted from UV region to longer wavelength compared with the pure tungstates and molybdates. Thus, the developed products can be perfectly excited by NUV or blue light. And some impressive achievements have been obtained in these strategies, such as Sr_2_ZnW_1−*x*_Mo_*x*_O_6_:Eu^3+^,Li^+^, La_3_BW_1−*x*_Mo_*x*_O_9_:Eu^3+^, NaLa(MoO_4_)_2−*x*_(WO_4_)_*x*_:Eu^3+^ and (Sr_*x*_Ba_1−*x*_)_2_CaMo_1−y_W_y_O_6_:Eu^3+^ ^32–35^. Obviously, most of the current researches mainly focus on the effect of transition metal (Mo^6+^ and W^6+^) ions on the luminescent properties of Eu^3+^-activated molybdates-tungstates compounds, while the influence of Eu^3+^ ion concentration on their luminescent performance is barely investigated.

Recently, the La_2_MoO_6_ has been intensively studied as the luminescent host material on account of its intrinsic luminescent properties and high stability^36,37^. Furthermore, it was also found that the La_2_WO_6_ had broad absorption band in the UV region^38^. However, these RE ions activated La_2_MoO_6_ or La_2_WO_6_ phosphors can only be efficiently excited by the deep UV light which makes them insufficient for NUV chip-based WLEDs. To figure out this shortage and improve their optical performance, the W^6+^ ions were introduced into the La_2_MoO_6_ host lattice and the La_2_Mo_0.6_W_0.4_O_6_ (La_2_MoO_6_-La_2_WO_6_) was produced. In present work, a facile citrate-assisted sol-gel route was applied to prepare the Eu^3+^-activated La_2_MoO_6_-La_2_WO_6_ red-emitting phosphors. The phase structure, morphology, lifetime, luminescent behaviors and thermal stability of the final products were detailedly studied. In addition, a theoretical calculation based on the Judd-Ofelt theory was also carried out to analyze the local crystal environment surrounding the Eu^3+^ ions. Finally, to clarify the applicability of resultant compounds for indoor lighting, a WLED device was implemented by utilizing an NUV LED chip and a mixture of synthesized red-emitting phosphors, commercial blue-emitting and green-emitting phosphors.

## Results and Discussion

**Figure 1:**
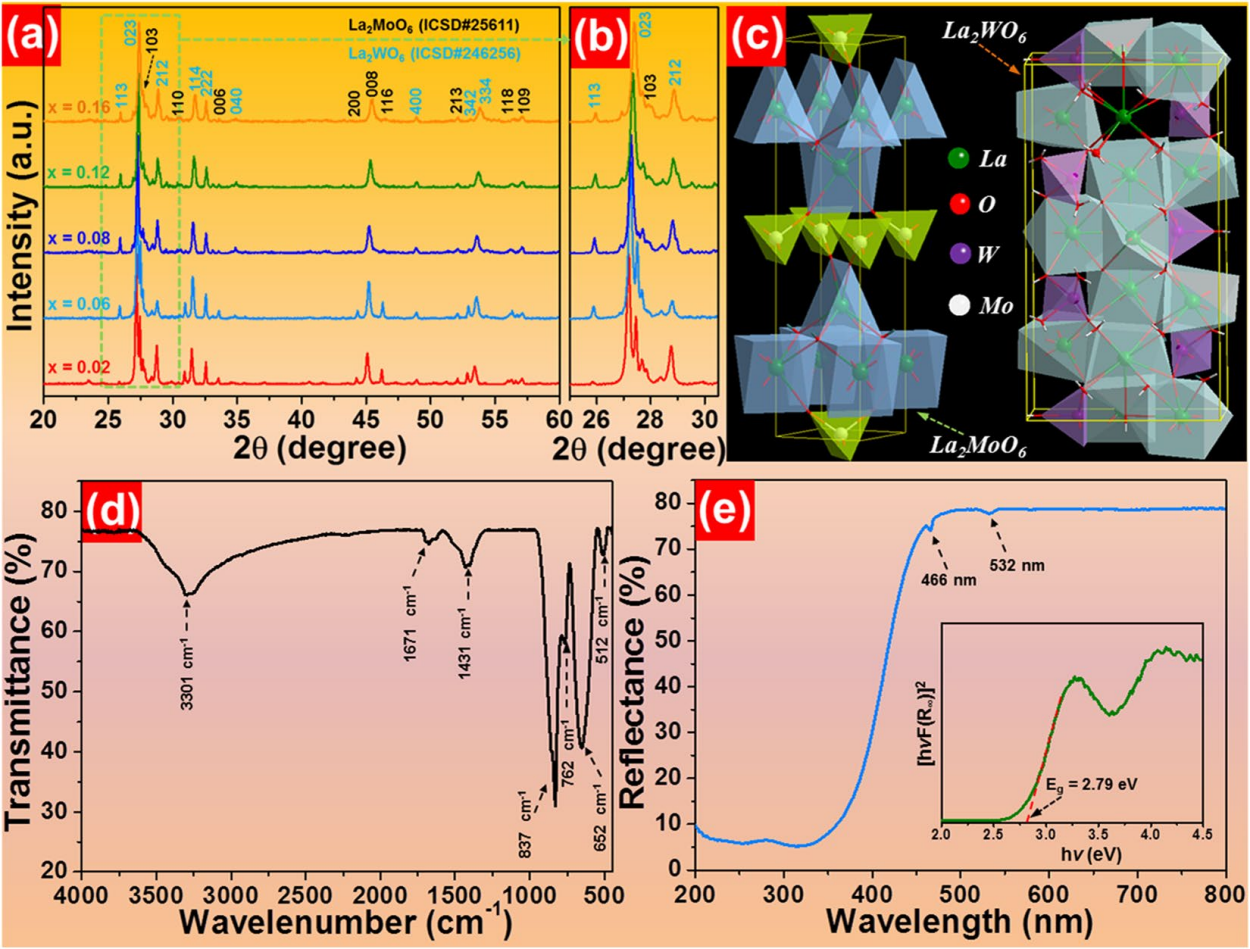
(**a**) Representative XRD patterns of La_2_MoO_6_-La_2_WO_6_:2*x*Eu^3+^ (*x* = 0.02, 0.06, 0.08, 0.12 and 0.16) red-emitting phosphors sintered at 850 °C. (**b**) Magnified XRD patterns in the 2θ range of 25–30.5°. (**c**) Crystal structures of La_2_MoO_6_ and La_2_WO_6_. (**d**) FTIR spectrum and (**e**) diffuse reflectance spectrum of the La_2_MoO_6_-La_2_WO_6_:0.24Eu^3+^ red-emitting phosphors. Inset depicts the calculation of band gap of the resultant samples utilizing Kubellka-Munk function.

The phase compositions of the final products were identified by X-ray diffraction (XRD). From the XRD patterns (see Fig. 1(a)), it is evident that all the samples exhibit similar diffraction patterns. According to previous literatures^28,39,40^, one knows that the resultant compounds consist of the mixed phases of La_2_MoO_6_ (ICSD#25611) and *β*-La_2_WO_6_ (ICSD#246256), revealing that the Eu^3+^ ions are incorporated into the host lattices and Eu^3+^-activated La_2_MoO_6_-La_2_WO_6_ red-emitting phosphors are successfully prepared. Moreover, with the increase of Eu^3+^ ion concentration, the diffraction peaks shift to larger angle which is attributed to inconsistent ionic radii between the Eu^3+^ and La^3+^ ions, as displayed in Fig. 1(b). The unit cell crystal structures of La_2_MoO_6_ and La_2_WO_6_ are presented in Fig. 1(c). As disclosed, in La_2_MoO_6_, the La^3+^ ions are surrounded by six oxygen atoms and the Mo^6+^ ions are coordinated with four oxygen atoms. In comparison, the La^3+^ ions in the La_2_WO_6_ are surrounded by eight oxygen atoms and the W^6+^ ions are coordinated with four oxygen atoms. Figure 1(d) shows the FTIR spectrum of La_2_MoO_6_-La_2_WO_6_:0.24Eu^3+^ red-emitting phosphors in the range of 450–4000 cm^−1^. The absorption peaks centered at around 3301 and 1671 cm^−1^ are associated to the O-H symmetric stretching vibration^16,41^. The absorption band located at around 1431 cm^−1^ is ascribed to the H-O-H blending vibration^42^. Furthermore, the intense absorption bands at about 837, 762, 652 and 512 cm^−1^ are related to the Mo-O-Mo, W-O-W, W-O and Mo-O stretching vibration modes, respectively^41,43,44^.

The diffuse reflectance spectrum of the La_2_MoO_6_-La_2_WO_6_:0.24Eu^3+^ red-emitting phosphors was recorded as shown in Fig. 1(e). As demonstrated, the studied samples possess strong absorption in the NUV region corresponding to the absorption of the host material which coincides well with the excitation spectrum. Meanwhile, two narrow bands located at approximately 466 and 532 nm originating from the characteristic absorption of Eu^3+^ ions are also observed^44^. As is known, the diffuse reflectance spectrum can be converted to the Kubelka-Munk function (*F*(*R*_∞_)) with the help of following formula^45^:

1 $$F({R}_{\infty })=\frac{{(1-R)}^{2}}{2R}=\frac{k}{s},$$

where *R* is the sample reflectivity, *k* denotes the molar absorption constant of the compound and *s* stands for the scattering coefficient. Moreover, the relationship between the optical band gap and absorption coefficient of luminescent materials can be roughly expressed as^44,45^:

2 $$\alpha hv\approx A{(hv-{E}_{g})}^{1/2}.$$

In this equation, *α*, *hv*, *E*_g_ and *A* present the optical absorption coefficient, phonon energy, band gap and proportionality coefficient, respectively. Combined with Eqs (1) and (2), the following expression is achieved:

3 $${[hvF({R}_{\infty })]}^{2}=B(hv-{E}_{g}).$$

To estimate the optical band gap of the La_2_MoO_6_-La_2_WO_6_:0.24Eu^3+^ red-emitting phosphors, the plot of [*hvF*(*R*_∞_)]^2^ versus *hv* is drawn, as presented in the inset of Fig. 1(e). As demonstrated (see the inset of Fig. 1(e)), the optical band gap is determined to be about 2.79 eV by deducing the linear fitted region to [*hvF*(*R*_∞_)]^2^ = 0.

**Figure 2:**
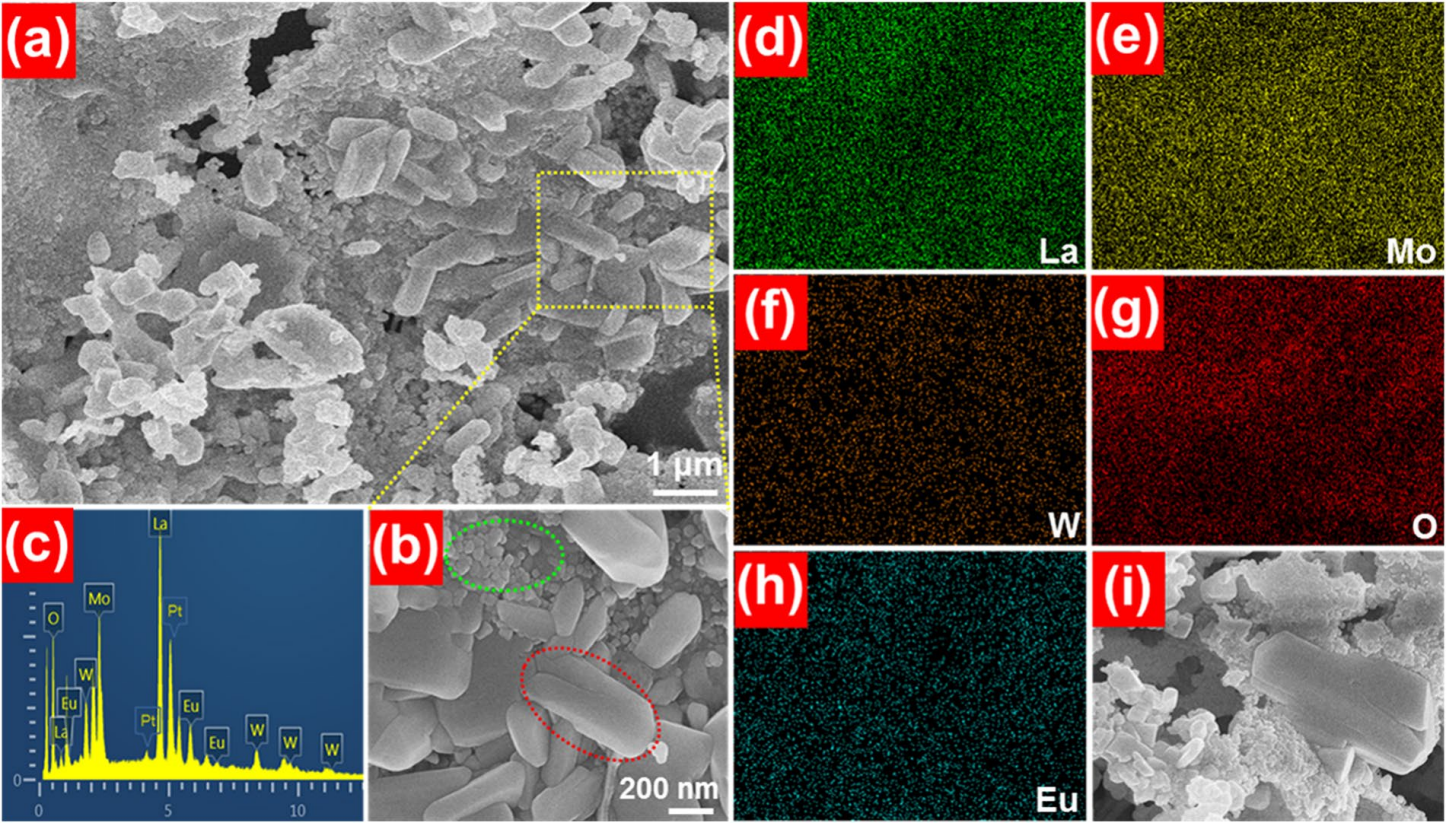
(**a** and **b**) FE-SEM images, (**c**) EDX spectrum and (**d**–**i**) elemental mapping of the La_2_MoO_6_-La_2_WO_6_:0.24Eu^3+^ red-emitting phosphors.

The microstructure and morphological properties of the prepared samples are characterized by FE-SEM. From the FE-SEM images, as described in Fig. 2(a) and (b), the synthesized compounds are made up of two different sized particles including small nanoparticles with the size around 70 nm and large nanoparticles with the average size about 600 nm, further indicating that the resultant samples consist of the hybrid phases of La_2_MoO_6_ and La_2_WO_6_. The EDX spectrum shown in Fig. 2(c) reveals the presence of La, Mo, W, O and Eu in the prepared samples. Furthermore, the occurrence of Pt peak in the EDX spectrum is assigned to the platinum electrode for measuring the FE-SEM image. In addition, the elemental mapping result suggests that the elements presented in the studied samples are homogeneously distributed (see Fig. 2(d–i)). These characteristics further verify the successful formation of Eu^3+^-activated La_2_MoO_6_-La_2_WO_6_ red-emitting phosphors, which coincides well with the deduction obtained from the XRD pattern.

**Figure 3:**
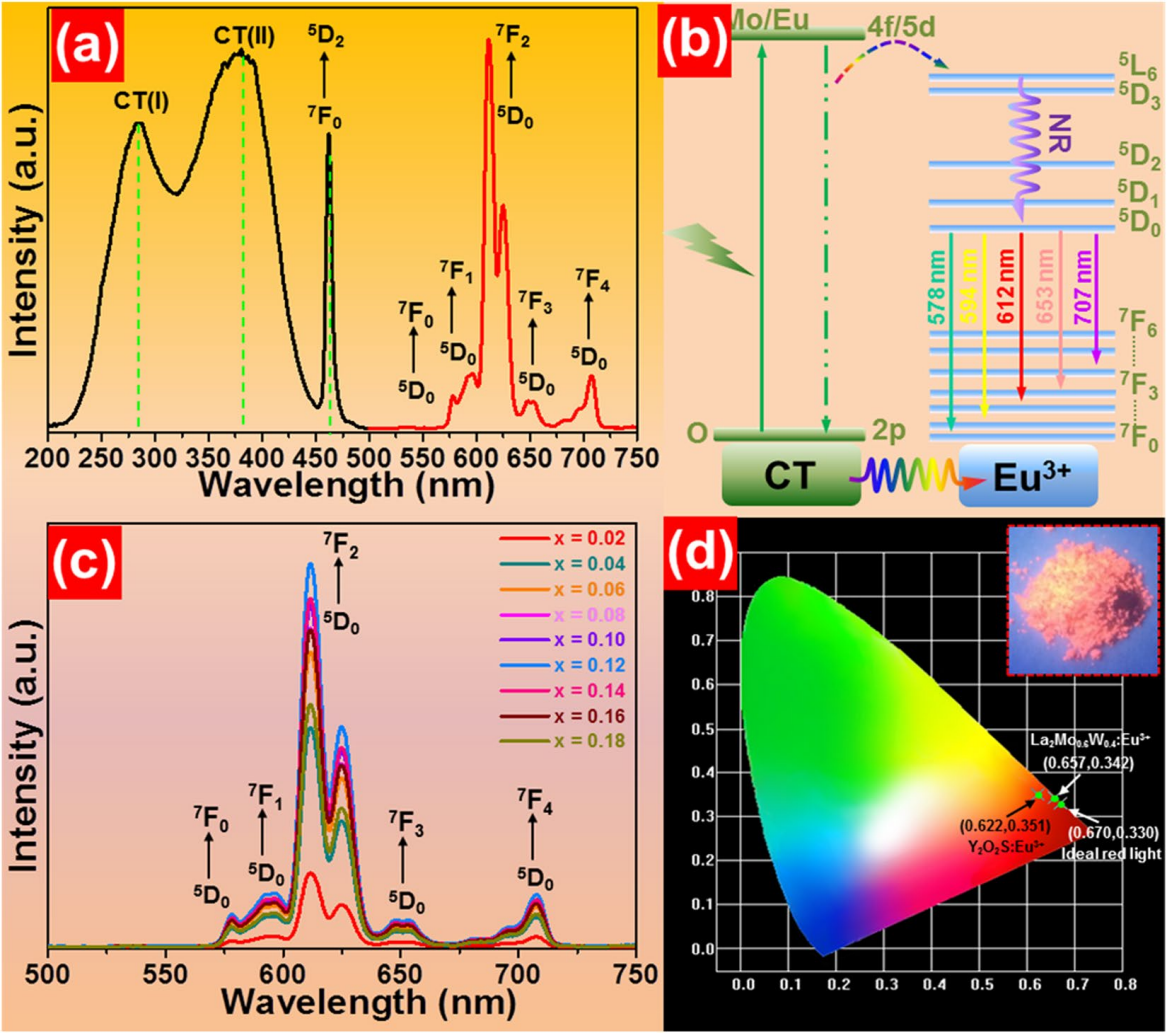
(**a**) PL excitation (λ_em_ = 612 nm) and emission (λ_ex_ = 379 nm) spectra of the La_2_MoO_6_-La_2_WO_6_:0.24Eu^3+^ red-emitting phosphors. (**b**) Simplified energy level diagram as well as the luminescent processes in the Eu^3+^-activated La_2_MoO_6_-La_2_WO_6_ compounds. (**c**) PL emission spectra of the La_2_MoO_6_-La_2_WO_6_:2*x*Eu^3+^ red-emitting phosphors as a function of Eu^3+^ ion concentration. (**d**) CIE chromaticity diagram of La_2_MoO_6_-La_2_WO_6_:0.24Eu^3+^ red-emitting phosphors. Inset shows the luminescent image excited at 365 nm of LED lamp.

The photoluminescence (PL) excitation spectrum of the La_2_MoO_6_-La_2_WO_6_:0.24Eu^3+^ red-emitting phosphors monitored at the emission wavelength of Eu^3+^ ions (612 nm) is described in Fig. 3(a). Obviously, the excitation spectrum consists of two ultrabroad absorption bands in the UV/NUV region and a narrow peak in the blue region. The first broad band, which is marked as CT(I), ranging from 220 to 320 nm centered at around 283 nm is assigned to the charger transfer from the oxygen ligands to tungsten ions^12,46^. Moreover, the second broad band, named as CT(II), with a central wavelength of 379 nm, which matches well with the commercial NUV LED chip, is related to the overlapped transitions of O^2−^ → Mo^6+^ and O^2−^ → Eu^3+^ ^28,32^. The sharp absorption band at 462 nm is ascribed to the ^7^F_0_ → ^5^D_2_ transition of Eu^3+^ ions^33^. Furthermore, the excitation spectral profiles are slightly varied with the increase of Eu^3+^ ion concentration, as depicted in Fig. S1. Note that, in comparison with other peaks, the excitation band located at 379 nm exhibits the strongest intensity, demonstrating that the studied samples can be efficiently excited by NUV light which is helpful for its application in solid-state lighting. Upon the irradiation of 379 nm light, the PL emission spectrum presented in Fig. 3(a) is dominated by an intense red emission at about 612 nm arising from the ^5^D_0_ → ^7^F_2_ transition of Eu^3+^ ions^5,42^. Apart from the strong red emission, four weak emission peaks situated at approximately 578, 594, 650 and 708 nm which are attributed to the ^5^D_0_ → ^7^F_0_, ^5^D_0_ → ^7^F_1_, ^5^D_0_ → ^7^F_3_ and ^5^D_0_ → ^7^F_4_ intra-configurational transitions of Eu^3+^ ions, respectively are also detected in the emission spectrum^12,21^. It is widely accepted that the yellow (^5^D_0_ → ^7^F_1_) and red (^5^D_0_ → ^7^F_2_) emissions are two featured emissions of Eu^3+^ ions. In particular, the ^5^D_0_ → ^7^F_1_ transition is regarded as the magnetic dipole transition (∆*J* = 0, ±1) which is insensitive to the crystal environment surrounding the Eu^3+^ ions, whereas the red emission corresponding to the ^5^D_0_ → ^7^F_2_ transition pertains to the hypersensitive electric dipole transition (∆*J* ≤ 6, when *J* or *J*′ = 0, ∆*J* = 2, 4, 6) and its intensity is largely dependent on the crystal field around the Eu^3+^ ions. In general, the ^5^D_0_ → ^7^F_2_ transition prevails in the luminescent spectrum when the Eu^3+^ ions occupy the sites with non-inversion symmetry, while the yellow emission (^5^D_0_ → ^7^F_2_) becomes the strongest one when the Eu^3+^ ions are located at symmetric cation circumstance^28,47^. As described in Fig. 3(a), it is evident that the emission intensity of the ^5^D_0_ → ^7^F_2_ transition is much higher than that of the ^5^D_0_ → ^7^F_1_ transition, demonstrating that the Eu^3+^ ions occupy the positions with low symmetry and non-inversion center in the host lattices. In addition, with the help of Judd-Ofelt theory, the optical transition intensity parameters, Ω_2_ and Ω_4_, were calculated to better comprehend the local structure environment surrounding the Eu^3+^ ions. The values of Ω_2_ and Ω_4_ are determined to be about 6.8 × 10^−20^ and 1.2 × 10^−20^ cm^2^, respectively, (see Supplementary Information), further verifying that the Eu^3+^ ions take up the low symmetry sites.

The three-dimensional (3D) PL emission spectra and contour lines of the La_2_MoO_6_-La_2_WO_6_:0.24Eu^3+^ red-emitting phosphors were measured in the excitation wavelength range of 220–420 nm, as depicted in Fig. S2(a) and (b), respectively. As presented in Fig. S2(b), the contour lines exhibit the characteristic emissions of Eu^3+^ ions originating from the ^5^D_0_ excited level to the ^7^F_0_, ^7^F_1_, ^7^F_2_, ^7^F_3_ and ^7^F_4_ ground states. Furthermore, both the 3D emission spectra and contour lines reveal the intense emission intensities between 270 and 400 nm excitation wavelengths, suggesting that the resultant products can be pumped by UV/NUV light which is in good agreement with the deduction achieved from the excitation spectrum. The result also confirms that the NUV LED chips are the efficient pumping sources for the La_2_MoO_6_-La_2_WO_6_:2*x*Eu^3+^ red-emitting phosphors which make them suitable for solid-state lighting. In order to expound the involved luminescent mechanism in the La_2_MoO_6_-La_2_WO_6_:2*x*Eu^3+^ system, the simplified energy level diagram as well as the proposed luminescent processes is displayed in Fig. 3(b). In brief, upon UV/NUV light excitation, the incident photons are absorbed by the host lattices. Subsequently, the energy is transferred to the adjacent Eu^3+^ ions and the ^5^L_6_ level is populated. Then, the electrons located at the ^5^L_6_ level decay to the ^5^D_0_ excited level by means of nonradiative (NR) transition. Ultimately, the emissions of Eu^3+^ ions are generated through the radiative transitions of ^5^D_0_ → ^7^F_*J*_ (*J* = 0, 1, 2, 3, 4), as described in Fig. 3(b).

As we know, the luminescent performance of the RE ions activated materials is greatly dependent on the dopant concentration. For the purpose of exploring the optimal doping concentration of Eu^3+^ ions in the La_2_MoO_6_-La_2_WO_6_ host lattices, a series of Eu^3+^-activated La_2_MoO_6_-La_2_WO_6_ compounds were prepared and their luminescent behaviors were studied in detail. Figure 3(c) describes the PL emission spectra of the La_2_MoO_6_-La_2_WO_6_:2*x*Eu^3+^ samples as a function of Eu^3+^ ion concentration. Obviously, all of the compounds emit the specific emissions of Eu^3+^ ions and the spectral emission profiles are barely changed with raising the doping concentration except the emission intensity. From the doping concentration-dependent PL emission intensity curve, as demonstrated in Fig. S3(a), it is clear that the emission intensity increases sharply with the increment of Eu^3+^ ion concentration and the optimum doping concentration is found to be 12 mol%. However, with further addition of the Eu^3+^ ions, the concentration quenching phenomenon occurs, which is associated with NR energy transfer among the dopants. Generally, the NR energy transfer among the dopants can be realized by means of radiation reabsorption and electric multipolar interaction. As presented in Fig. 3(a), there are no any overlaps between the excitation and emission spectra, revealing that the NR energy transfer among the Eu^3+^ ions is not caused by the radiation reabsorption. Herein, the NR energy transfer among the Eu^3+^ ions should be controlled by electric multipolar interaction. On the basis of Dexter theory, the following expression is given^48^:

4 $$\frac{I}{x}=k{[1+\beta {(x)}^{\theta /3}]}^{-1},$$

where *I* is the emission intensity, *x* denotes the doping concentration, *k* and *β* are constants, and *θ* = 6, 8 and 10 are related to dipole-dipole, dipole-quadrupole and quadrupole-quadrupole interactions, respectively. The plot of log(*I*/*x*) versus log(*x*) is shown in Fig. S3(b). As displayed, the experimental data can be linearly fitted with a slope of −1.85 and thus, the *θ* value is calculated to be 5.55 which approaches to 6, confirming that the concentration quenching of Eu^3+^ ions in the La_2_MoO_6_-La_2_WO_6_:2*x*Eu^3+^ compounds is dominated by dipole-dipole interaction.

The Commission International de I’Eclairage (CIE) chromaticity coordinate of Eu^3+^-activated La_2_MoO_6_-La_2_WO_6_ red-emitting phosphors with optimal doping concentration was calculated, as described in Fig. 3(d). The estimated CIE chromaticity coordinate (0.657, 0.342) is located in the edge of red region which is very close to the ideal red light (0.670, 0.330), and outclasses that of the commercial Y_2_O_2_S:Eu^3+^ (0.622, 0.351) red-emitting phosphors. Meanwhile, under the irradiation of NUV light, the synthesized samples emit dazzling red emissions (see the inset of Fig. 3(d)). Apart from the color coordinate, the color purity is another essential parameter to evaluate the chromatic properties of the resultant phosphors which can be defined as^12,42^:

5 $$Color\,\,purity=\frac{\sqrt{{(x-{x}_{i})}^{2}+{(y-{y}_{i})}^{2}}}{\sqrt{{({x}_{d}-{x}_{i})}^{2}+{({y}_{d}-{y}_{i})}^{2}}}\times 100 \% ,$$

where (*x*, *y*), (*x*_i_, *y*_i_) and (*x*_*d*_, *y*_*d*_) denote the color coordinates of the studied samples, white illumination and dominate wavelength, respectively. Herein, (*x*, *y*) = (0.657, 0.342), (*x*_i_, *y*_i_) = (0.310, 0.316) and (*x*_*d*_, *y*_*d*_) = (0.672, 0.328). As a consequence, the color purity of red emission is determined to be as high as 96.1% which is much superior to previous reports, such as CaMo_0.6_W_0.4_O_4_:Eu^3+^ (93.8%) and SrMoO_4_:Eu^3+^ (85.8%)^12,49^. These results demonstrate that the Eu^3+^-activated La_2_MoO_6_-La_2_WO_6_ red-emitting phosphors with high color purity, good color coordinate as well as superior luminescent properties may have promising applications in solid-state lighting.

**Figure 4:**
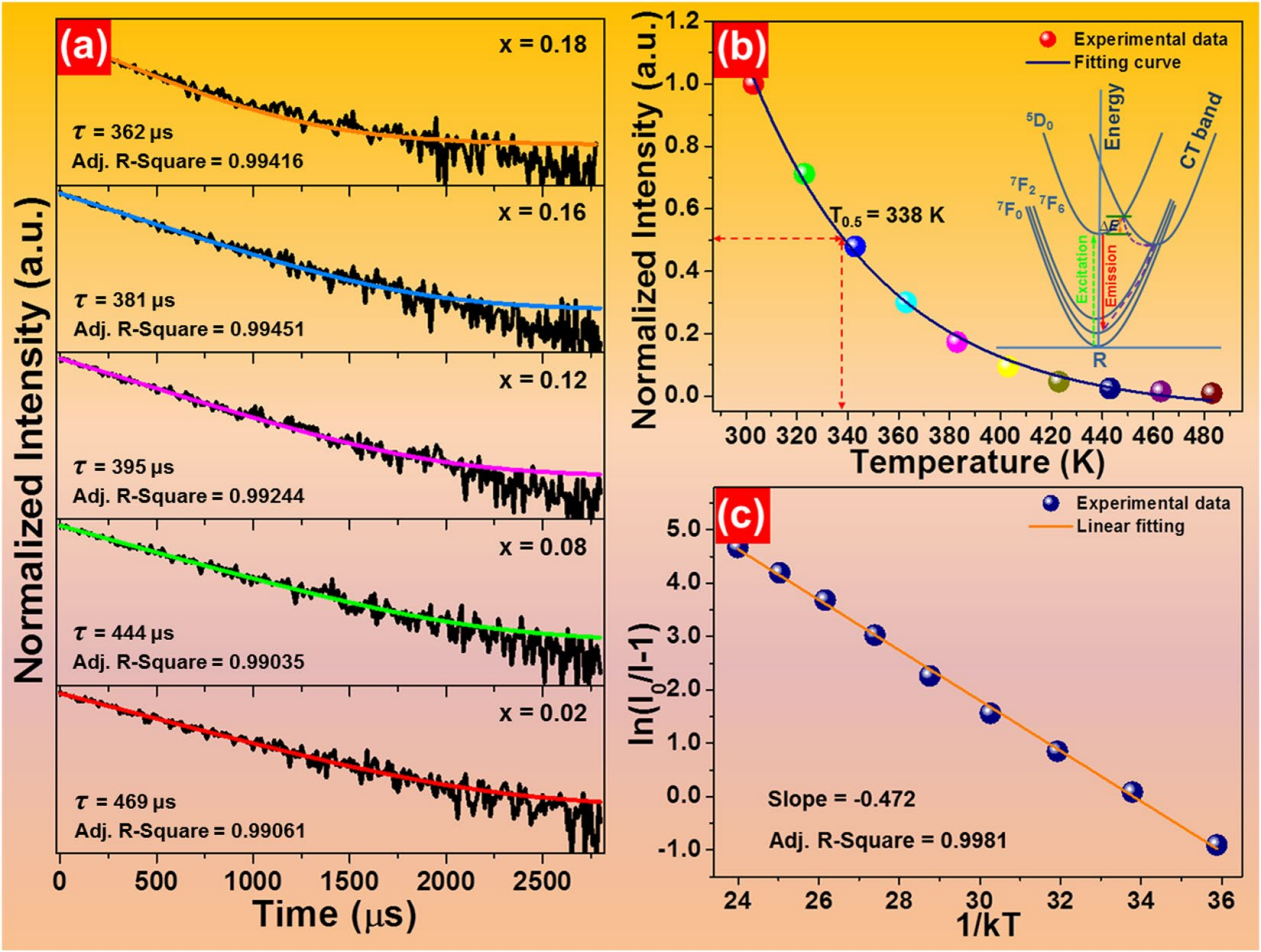
(**a**) Representative decay curves of the La_2_MoO_6_-La_2_WO_6_:2*x*Eu^3+^ (*x* = 0.02, 0.08, 0.12, 0.16 and 0.18) red-emitting phosphors. (**b**) PL emission intensity as a function of temperature. (**c**) Plot of ln(*I*_0_/*I*−1) versus 1/*kT*. Inset shows the possible channels for the thermal quenching behavior of the studied samples.

For the sake of understanding the luminescence dynamics, the room-temperature decay curves of the final products were recorded. Figure 4(a) shows the representative decay curves of the La_2_MoO_6_-La_2_WO_6_:2*x*Eu^3+^ (*x* = 0.02, 0.08, 0.12, 0.16 and 0.18) red-emitting phosphors (λ_ex_ = 379 nm, λ_em_ = 612 nm). As presented, the recorded curves can be perfectly fitted with a single-exponential decay model, as defined below:

6 $$I(t)={I}_{0}+A\,\exp (-t/\tau ),$$

where *I*(*t*) and *I*_0_ refer to the emission intensities at times *t* and *t* = 0, *A* is constant and *τ* denotes the lifetime. According to the fitting results, the measured lifetime is found to be 469, 444, 395, 381 and 362 μs, respectively when the Eu^3+^ ion concentration is 2, 8, 12, 16 and 18 mol%. Clearly, the decay time exhibits a tendency of decrease with the increase of doping concentration, suggesting the existence of NR energy transfer and concentration quenching in Eu^3+^ ions-based compounds. As is known, with the introduction of Eu^3+^ ions, the distance between the dopants will be decreased, leading to the enhanced NR energy transfer possibility between the Eu^3+^ ions as well as the decreased lifetime.

For verifying the feasibility of the synthesized phosphors for solid-state lighting application, their thermal stability should be evaluated since it can vastly affect the light output, working lifetime and CRI of the LED device. Under 379 nm light excitation, the temperature-dependent PL emission spectra of La_2_MoO_6_-La_2_WO_6_:0.24Eu^3+^ red-emitting phosphors were measured as presented in Fig. S4. It can be seen that the emission peaks scarcely changed with raising the temperature from 303 to 483 K which is beneficial to achieve stable emission color at high temperature. In comparison, the emission intensity decreases rapidly with the increment of temperature owing to the NR phonon relaxation from the high populated energy level *via* crossover process and the possible channels for the thermal quenching are shown in the inset of Fig. 4(b) ^25^. Furthermore, the relatively small optical band gap may also be reasonable for the sharply deceased emission intensity with the elevated temperature. The thermal quenching temperature (T_0.5_), defined as the temperature at which the emission intensity decreases to half of its initial value, is determined to be about 338 K (see Fig. 4(b)). In order to better understand the thermal quenching phenomenon, the following expression is employed to evaluate the activation energy^50,51^:

7 $$\mathrm{ln}(\frac{{I}_{0}}{I}-1)=\,\mathrm{ln}\,A-\frac{{\rm{\Delta }}E}{kT}.$$

In this expression, *I*_0_ is the initial emission intensity, *I* refers to the emission intensity at different temperatures, *A* is constant, *k* is Boltzmann constant and ∆*E* is the activation energy from ^5^D_0_ level to the CT band. According to the plot of ln(*I*_0_/*I*−1) versus 1/*kT*, as presented in Fig. 4(c), the recorded data are linearly fitted with a slope of −0.472. Therefore, the activation energy for the thermal quenching is determined to be 0.472 eV.

Apart from the chromatic properties, color purity and thermal stability, the quantum efficiency of the resultant phosphors is another vital factor to identify their suitability for solid-state lighting application. As is known, the quantum efficiency of the Eu^3+^ ions activated phosphors can be estimated by means of the emission spectrum and lifetime, which was proposed by Kodaira *et al*. and subsequently applied by other researchers^52–54^. According to the previous report by Kodaira *et al*., the following expressions can be achieved^52^:

8 $${A}_{0-J}={A}_{0-1}\frac{{I}_{0-J}}{{I}_{0-1}}\frac{h{v}_{0-1}}{h{v}_{0-J}},$$

9 $${A}_{rad}=\sum _{J=0,1,2,3,4}{A}_{0-J},$$

10 $$\frac{1}{\tau }={A}_{rad}+{A}_{nrad},$$

11 $$\eta =\frac{{A}_{rad}}{{A}_{rad}+{A}_{nrad}}.$$

In these expressions, *A*_*0-J*_ stands for the Einstein coefficient of spontaneous emission corresponding to the ^5^D_0_ → ^7^F_*J*_ transitions. *A*_*rad*_ and *A*_*nrad*_ show the radiative and non-radiative transition rates, respectively, *I*_*0-J*_ is the integrated emission intensities of ^5^D_0_ → ^7^F_*J*_ transitions, *hv*_*0-J*_ exhibits the energy of the ^5^D_0_ → ^7^F_*J*_ transitions and *τ* is the decay time. Since the ^5^D_0_ → ^7^F_1_ transition belongs to the magnetic transition and it is independent of the crystals field^55^, the value of *A*_*0*-*1*_ is determined to be approximately 50 s^−1^. From the recorded decay curve (see Fig. 4(a)), one knows that the lifetime of the La_2_MoO_6_-La_2_WO_6_:0.24Eu^3+^ red-emitting phosphors is 395 μs. As a result, with the help of Eqs (8–11), the quantum efficiency of the Eu^3+^-activated La_2_MoO_6_-La_2_WO_6_ red-emitting phosphors with the optimal doping concentration is calculated to be 27.7%. It is evident that the achieved value is comparable with other reported red-emitting phosphors, in which their quantum efficiencies were also estimated by utilizing the above method, such as (Ca,Sr)(Mo,W)O_4_:Eu^3+^ (28.6%), Ca_3_Sn_3_Nb_2_O_14_:Eu^3+^ (30.29%), La_2_W_1.6_Mo_0.4_O_9_:Eu^3+^ (22.38%) and SrNb_2_O_6_:Eu^3+^ (17.84%)^56–59^, further implying that the resultant red-emitting phosphors are promising for WLEDs. Additionally, to further understand the optical performance of the resultant phosphors, the quantum efficiency of the La_2_MoO_6_-La_2_WO_6_:0.24Eu^3+^ red-emitting phosphors was measured. Under 379 nm of excitation, the quantum efficiency of the studied phosphors is determined to be about 8.9% which is smaller than the theoretical value (27.7%). Similar phenomenon was reported in Eu^3+^-activated KYP_2_O_7_ red-emitting phosphors^54^. As is known, the quantum efficiency is measured for a special excitation wavelength, while the theoretically estimated quantum efficiency is greatly dependent on the *A*_0–1_ parameter which has to be fixed at a reasonable value of the characteristics of the Eu^3+^ ions in similar compounds^54^, resulting in the difference between the measured quantum efficiency and theoretically calculated quantum efficiency.

**Figure 5:**
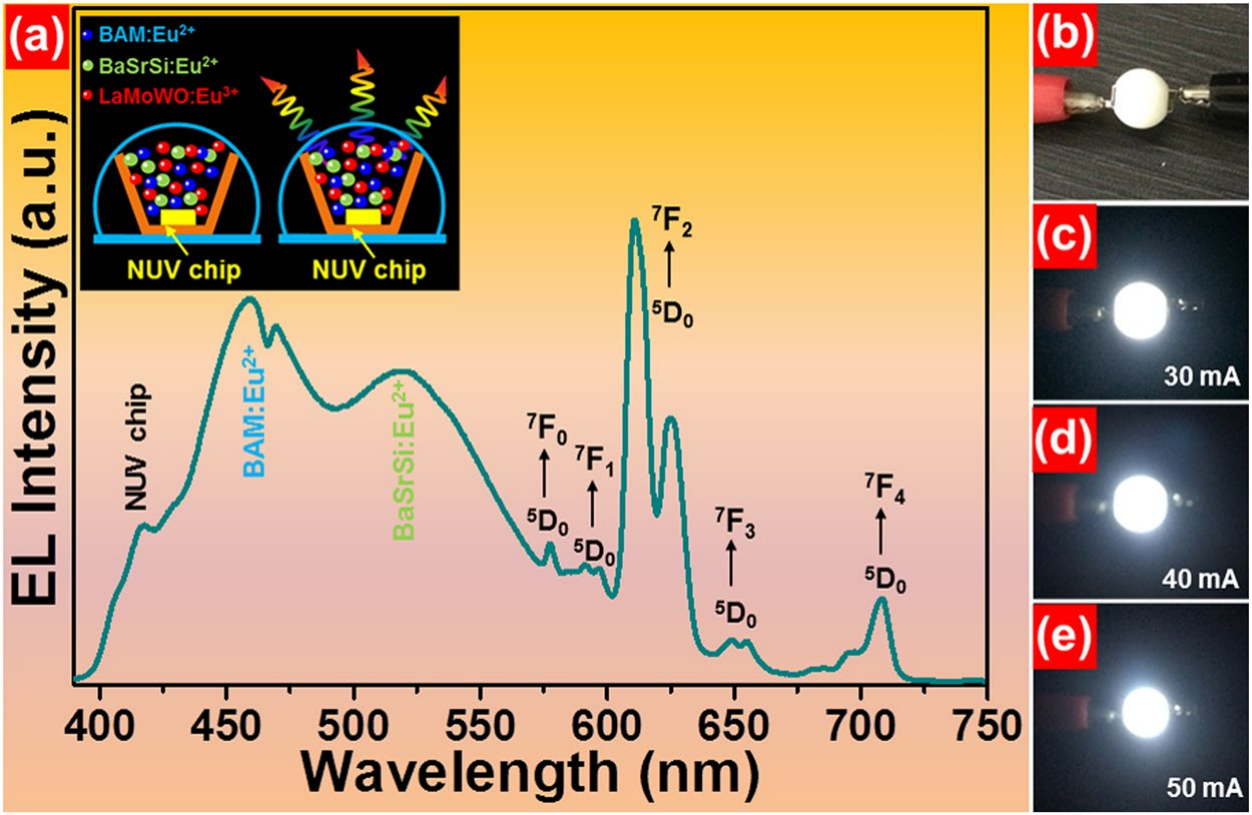
(**a**) EL emission spectrum of the fabricated WLED device under different operating currents. (**b**) Fabricated WLED device. (**c**–**e**) Digital luminescent images of the WLED device at different input currents of 30, 40 and 50 mA, respectively. Inset shows the schematic diagrams of the packaged WLED device.

To better confirm the suitability of the resultant phosphors for solid-state lighting application, a red-emitting LED device was fabricated by coating the La_2_MoO_6_-La_2_WO_6_:0.24Eu^3+^ red-emitting phosphors onto the surface of a NUV LED chip and its EL emission spectrum is shown in Fig. 5S(a). As disclosed, the EL emission spectrum consists of an intense peak located at around 412 nm originating from the NUV LED chip and several sharp emission bands in the wavelength range of 575–725 nm corresponding to the featured emissions of Eu^3+^ ions. Meanwhile, under a forward bias current of 50 mA, the fabricated LED device emits glaring red emission that can be seen by naked eyes (see Fig. S5(b) and (c)), suggesting that the Eu^3+^-activated La_2_MoO_6_-La_2_WO_6_ phosphors are promising candidates for red-emitting phosphors for indoor lighting. As a proof of the above deduction, a WLED device was prepared by integrating an NUV LED chip and a mixture of BaMgAl_10_O_17_:Eu^2+^ (BAM:Eu^2+^) blue-emitting phosphors, (Ba,Sr)_2_SiO_4_:Eu^2+^ (BaSrSi:Eu^2+^) green-emitting phosphors and La_2_MoO_6_-La_2_WO_6_:0.24Eu^3+^ red-emitting phosphors. The EL emission spectrum of the fabricated WLED device, which was driven by a forward bias current of 50 mA, was recorded, as shown in Fig. 5(a). Clearly, the EL emission spectrum can be divided into four parts, that is, an emission peak situated at 412 nm arising from the NUV LED chip, two broad emission bands centered at 461 and 521 nm originating from the commercial blue-emitting and green-emitting phosphors, respectively, and a series of narrow emission peaks are attributed to the characteristic emissions of Eu^3+^ ions in the La_2_MoO_6_-La_2_WO_6_:0.24Eu^3+^ red-emitting phosphors. Furthermore, the CCT and CRI values of the designed WLEDs device are 6492 K and 84.6, respectively. Figure 5(b) shows the fully packaged WLED device. Under different forward bias currents, the packaged WLEDs device emits dazzling white light and the emitting color barely changes with increasing the forward bias current, as displayed in Fig. 5(c–e). These characteristics make the Eu^3+^-activated La_2_MoO_6_-La_2_WO_6_ phosphors suitable for WLEDs as red-emitting phosphors.

## Conclusion

In summary, a series of novel Eu^3+^-activated La_2_MoO_6_-La_2_WO_6_ red-emitting phosphors were synthesized via a facile citrate-assisted sol-gel route. The resultant compounds possessed an ultrabroad intense excitation band from 220 to 450 nm and emitted glaring red emission with high color purity of 96.1% under 379 nm light excitation. The Eu^3+^ doping concentration in La_2_MoO_6_-La_2_WO_6_ host lattices was optimized as 12 mol% and the dipole-dipole interaction is revealed to contribute to the concentration quenching. Both the emission spectrum and theoretical calculation confirm that the Eu^3+^ ions occupy the low symmetry sites in the host lattices. The thermal stability of the prepared phosphors was characterized by temperature-dependent emission spectra and the activation energy is found to be 0.472 eV. The red-emitting LED device, which is made only with La_2_MoO_6_-La_2_MoO_6_:0.24Eu^3+^ phosphors, demonstrates that the synthesized compounds are suitable for indoor lighting applications. Additionally, the WLEDs packaged by a NUV LED chip, La_2_MoO_6_-La_2_MoO_6_:0.24Eu^3+^ red-emitting phosphors, commercial blue-emitting and green-emitting phosphors emitted dazzling white light with high CRI (84.6) and proper CCT (6492 K), further indicating that the Eu^3+^-activated La_2_MoO_6_-La_2_WO_6_ compounds are promising red-emitting phosphors for solid-state lighting.

## Experimental Section

### Materials and Synthesis

The citrate-assisted sol-gel technique was employed to synthesize the Eu^3+^-activated La_2_MoO_6_-La_2_WO_6_ (La_2_MoO_6_-La_2_WO_6_:2*x*Eu^3+^; *x* = 0.02, 0.04, 0.06, 0.08, 0.10, 0.12, 0.14, 0.16 and 0.18) red-emitting phosphors. Stoichiometric amounts of La(NO_3_)_3_∙6H_2_O, (NH_4_)_6_Mo_7_O_24_∙4H_2_O, Na_2_WO_4_∙2H_2_O and Eu(NO_3_)_3_∙5H_2_O were weighted and dissolved into 200 ml of de-ionized water to form a transparent homogenous solution. Subsequently, citric acid acting as a complexing agent was added into the above mixture. Then, the solution was covered with a polyethylene lid and heated at 80 °C for 30 min with drastic mechanical stirring. Afterwards, the lid was removed from the beaker and the solution was gradually evaporated to generate a yellow wet-gel. Later, the wet-gel was dried in oven at 120 °C for 12 h, and the xerogel was obtained. Ultimately, the xerogel was put into an alumina crucible and calcined at 850 °C for 6 h to form the Eu^3+^-activated La_2_MoO_6_-La_2_WO_6_ phosphors.

### Material Characterization

The phase compositions of the studied samples were examined on a Bruker D8 Advance diffractometer. The morphological behaviors and elemental composition of the synthesized compounds were characterized by using a field-emission scanning electron microscope (FE-SEM) (LEO SUPRA 55, Carl Zeiss) equipped with an energy-dispersive X-ray (EDX) spectrometer. The luminescent spectra of the phosphors were recorded by utilizing a fluorescence spectrometer (Scinco FluroMate FS-2). The spectrofluorometer (Edinburgh FS5) attached with an integrating sphere coated with barium sulfate was employed to measure the quantum efficiency. The temperature ranging from 303 to 483 K was controlled by a temperature controlled stage (NOVA ST540). The Fourier transform infrared (FTIR) and diffuse reflectance spectra were recorded by using a Thermo Nicolet-570 FTIR spectrophotometer and V-670 (JASCO) UV-vis spectrophotometer, respectively. Under a forward bias current of 50 mA, the multi-channel spectroradiometer (OL 770) was used to monitor the electroluminescence (EL) spectrum.

## Electronic supplementary material

Supplementary Information

## References

[1] Yang C, Som S, Das S, Lu C (2017). Synthesis of Sr_2_Si_5_N_8_:Ce^3+^ phosphors for white LEDs via an efficient chemical deposition. Sci. Rep..

[2] Huang X (2014). Red phosphor converts white LEDs. Nat. Photon..

[3] Zhong J (2017). Synthesis and spectroscopic investigation of Ba_3_La_6_(SiO_4_)_6_:Eu^2+^ green phosphors for white light-emitting diodes. Chem. Eng. J..

[4] Shang M, Liang S, Qu N, Lian H, Lin J (2017). Influence of Anion/Cation Substitution (Sr^2+^  → Ba^2+^, Al^3+^  → Si^4+^, N^3−^ → O^2−^) on Phase Transformation and Luminescence Properties of Ba_3_Si_6_O_15_:Eu^2+^ Phosphors. Chem. Mater..

[5] Zhang N, Guo C, Zheng J, Su X, Zhao J (2014). Synthesis, electronic structures and luminescent properties of Eu^3+^ doped KGdTiO_4_. J. Mater. Chem. C.

[6] Xia Z, Meijerink A (2017). Ce^3+^-Doped garnet phosphors: composition modification, luminescence properties and applications. Chem. Soc. Rev..

[7] Chen J (2016). Site-Dependent Luminescence and Thermal Stability of Eu^2+^ Doped Fluorophosphate toward White LEDs for Plant Growth. ACS Appl. Mater. Interfaces.

[8] Xie W, Liu G, Dong X, Wang J, Yu W (2016). Doping Eu^3+^/Sm^3+^ into CaWO_4_:Tm^3+^, Dy^3+^ phosphors and their luminescence properties, tunable color and energy transfer. RSC Adv..

[9] Li G, Tian Y, Zhao Y, Lin J (2015). Recent progress in luminescence tuning of Ce^3+^ and Eu^2+^-activated phosphors for pc-WLEDs. Chem. Soc. Rev..

[10] Jia Z, Xia M (2016). Blue-green tunable color of Ce^3+^/Tb^3+^ coactivated NaBa_3_La_3_Si_6_O_20_ phosphor via energy transfer. Sci. Rep..

[11] Xia Z, Xu Z, Chen M, Liu Q (2015). Recent developments in the new inorganic solid-state LED phosphors. Dalton Trans..

[12] Huang X, Li B, Guo H, Chen D (2017). Molybdenum-doping-induced photoluminescence enhancement in Eu^3+^activated CaWO_4_ red-emitting phosphors for white light-emitting diodes. Dyes Pigments.

[13] Dhanaraj J, Jagannathan R, Tricedi DC (2013). Y_2_O_2_S:Eu^3+^ nanocrystals—synthesis and luminescent properties. J. Mater. Chem..

[14] Li YQ (2006). Luminescence properties of red-emitting M_2_Si_5_N_8_:Eu^2+^ (M = Ca, Sr, Ba) LED conversion phosphors. J. Alloys Compd..

[15] Pust P (2012). Narrow-band red-emitting Sr[LiAl_3_N_4_]:Eu^2+^ as a next-generation LED-phosphor material. Nat. Mater..

[16] Hussain SK, Yu JS (2017). Broad red-emission of Sr_3_Y_2_Ge_3_O_12_:Eu^2+^ garnet phosphors under blue excitation for warm WLED applications. RSC Adv..

[17] Fiaczyk K, Zych E (2016). On peculiarities of Eu^3+^ and Eu^2+^ luminescence in Sr_2_GeO_4_ host. RSC Adv..

[18] Huang X (2017). Broadband dye-sensitized upconversion: A promising new platform for future solar upconverter design. J. Alloys Compd..

[19] Wang X (2017). Influence of Doping and Excitation Powers on Optical Thermometry in Yb^3+^-Er^3+^ doped CaWO_4_. Sci. Rep..

[20] Li K, Shang M, Lian H, Lin J (2016). Recent development in phosphors with different emitting colors via energy transfer. J. Mater. Chem. C.

[21] Zhong J (2017). Red-emitting CaLa_4_(SiO_4_)_3_O:Eu^3+^ phosphor with superior thermal stability and high quantum efficiency for warm w-LEDs. J. Alloys Compd..

[22] Du P, Zhang P, Kang SH, Yu JS (2017). Hydrothermal synthesis and application of Ho^3+^-activated NaYbF_4_ bifunctional upconverting nanoparticles for *in vitro* cell imaging and latent fingerprint detection. Sens. Actuator B.

[23] Zhang Y, Xu J, Cui Q, Yang B (2017). Eu^3+^-doped Bi_4_Si_3_O_12_ red phosphor for solid state lighting: microwave synthesis, characterization, photoluminescence properties and thermal quenching mechanisms. Sci. Rep..

[24] Grigorjevaite J, Katelnikovas A (2016). Luminescence and Luminescence Quenching of K_2_Bi(PO_4_)(MoO_4_):Eu^3+^ Phosphors with Efficiencies Close to Unity. ACS Appl. Mater. Interfaces..

[25] Wang L (2016). High luminescent brightness and thermal stability of red emitting Li_3_Ba_2_Y_3_(WO_4_)_8_:Eu^3+^ phosphor. Ceram. Int..

[26] Behrh GK, Gautier R, Latouche C, Jobic S, Serier-Brault H (2016). Synthesis and Photoluminescence Properties of Ca_2_Ga_2_SiO_7_:Eu^3+^ Red Phosphors with an Intense ^5^D_0_ → ^7^F_4_ Transition. Inor. Chem..

[27] Janulevicius M (2016). A. Luminescence and luminescence quenching of highly efficient Y_2_Mo_4_O_15_:Eu^3+^ phosphors and ceramics. Sci. Rep..

[28] Du P, Guo Y, Lee SH, Yu JS (2017). Broad near-ultraviolet and blue excitation band induced dazzling red emissions in Eu^3+^-activated Gd_2_MoO_6_ phosphors for white light-emitting diodes. RSC Adv..

[29] Chien T, Yang J, Hwang C, Yoshimura M (2016). Synthesis and photoluminescence properties of red-emitting Y_6_WO_12_:Eu^3+^ phosphors. J. Alloys Compd..

[30] Litterscheid C (2016). Solid solution between lithium-rich yttrium and europium molybdate as new efficient red-emitting phosphors. J. Mater. Chem. C.

[31] Wang C, Ye S, Li Y, Zhang Q (2017). The impact of local structure variation on thermal quenching of luminescence in Ca_3_Mo_*x*_W_1−*x*_O_6_:Eu^3+^ solid solution phosphors. J. Appl. Phys..

[32] Li L (2016). Luminescence enhancement in the Sr_2_ZnW_1-*x*_Mo_*x*_O_6_:Eu^3+^, Li^+^ phosphor for near ultraviolet based solid state lighting. J. Alloys Compd..

[33] Huang J, Hou B, Ling H, Liu J, Yu X (2014). Crystal Structure, Electronic Structure, and Photoluminescence Properties of La_3_BW_1−*x*_Mo_*x*_O_9_:Eu^3+^ Red Phosphor. Inorg. Chem..

[34] Lu Z, Wanjun T (2012). Synthesis and luminescence properties of Eu^3+^-activated NaLa(MoO_4_)(WO_4_) phosphor. Ceram Int..

[35] Sletnes M, Valmalette JC, Grande T, Einarsrud MA (2016). Compositional dependence of the crystal symmetry of Eu^3+^-doped (Sr_*x*_Ba_1−*x*_)_2_CaW_y_Mo_1−y_O_6_ phosphors. J. Solid. State. Chem..

[36] Du P, Yu JS (2017). Near-ultraviolet light induced visible emissions in Er^3+^-activated La_2_MoO_6_ nanoparticles for solid-state lighting and non-contact thermometry. Chem. Eng. J..

[37] Ming F, Zhang X, Li H, Seo HJ (2012). Synthesis and spectral characteristics of La_2_MoO_6_:Ln^3+^ (Ln = Eu, Sm, Dy, Pr, Tb) polycrystals. J. Rare. Earth..

[38] Ishigaki T (2009). Melt synthesis of oxide red phosphors La_2_WO_6_:Eu^3+^. Physics Procedia.

[39] Allix M (2011). Synthesis and Structure Determination of the High Temperature Form of La_2_WO_6_. Cryst. Growth. Des.

[40] Chambrier M, Kodjikian S, Ibberson PM, Goutenoire F (2008). Ab-initio structure determination of *β*-La_2_WO_6_. J. Solid. State. Chem..

[41] Soni AK, Rai VK (2014). Intrinsic optical bistability and frequency upconversion in Tm^3+^-Yb^3+^-codoped Y_2_WO_6_ phosphor. Dalton Trans..

[42] Du P, Yu JS (2015). Photoluminescence and cathodoluminescence properties of Eu^3+^ ions activated AMoO_4_ (A = Mg, Ca, Sr, Ba) phosphors. Mater. Res. Bull..

[43] Nithya VD, Selvan RK, Vasylechko L, Sanjeeviraja C (2014). Surfactant assisted sonochemical synthesis of Bi_2_WO_6_ nanoparticles and their improved electrochemical properties for use in pseudocapacitors. RSC Adv..

[44] Wang L (2015). Dual-Mode Luminescence with Broad Near UV and Blue Excitation Band from Sr_2_CaMoO_6_:Sm^3+^ Phosphor for White LEDs. J. Phys. Chem. C.

[45] Zheng J (2015). An efficient blue-emitting Sr_5_(PO_4_)_3_Cl:Eu^2+^ phosphor for application in near-UV white light-emitting diodes. J. Mater. Chem. C.

[46] Wang L (2015). Photoluminescence properties, crystal structure and electronic structure of a Sr_2_CaWO_6_:Sm^3+^ red phosphor. RSC Adv..

[47] Wei Y (2017). Emitting-tunable Eu^(2+/3+)^-doped Ca_(8-*x*)_La_(2+*x*)_(PO_4_)_6-*x*_(SiO)_*x*_O_2_ apatite phosphor for n-UV WLEDs with high-color-rendering. RSC Adv..

[48] Dexter, D. L. A theory of sensitized luminescence in solid. *J. Chem. Phys*. **21**, 836-850 (1953).

[49] Du P, Yu JS (2015). Dual-enhancement of photoluminescence and cathodoluminescence in Eu^3+^-activated SrMoO_4_ phosphors by Na^+^ doping. RSC Adv..

[50] Chen Y (2016). Blue-emitting phosphor Ba_4_OCl_6_:Eu^2+^ with good thermal stability and a tiny chromaticity shift for white LEDs. J. Mater. Chem. C.

[51] Lv W (2014). Crystal Structure and Luminescence Properties of Ca_8_Mg_3_Al_2_Si_7_O_28_:Eu^2+^ for WLEDs. Adv. Opt. Mater..

[52] Kodaira CA, Brito HF, Malta OL, Serra OA (2003). Luminescence and energy transfer of the europium (III) tungstate obtained via the Pechini method. J. Lumin..

[53] Kumar A, Kumar J (2011). Perspective on europium activated fine-grained metal molybdate phosphors for solid state illumination. J. Mater. Chem..

[54] Pazik R, Watras A, Macalik L, Deren PJ (2014). One step urea assisted synthesis of polycrystalline Eu^3+^ doped KYP_2_O_7_ -luminescence and emission thermal quenching properties. New J. Chem..

[55] Sá GF (2000). Spectroscopic properties and design of highly luminescent lanthanide coordination complexes. Coord. Chem. Rev..

[56] Cao FB, Li LS, Tian YW, Chen YJ, Wu XR (2011). Investigation of red-emission phosphors (Ca,Sr)(Mo,W)O:Eu^3+^ crystal structure, luminous characteristics and calculation of Eu^3+ 5^D_0_ quantum efficiency. Thin. Solid. Film..

[57] Sreena TS (2015). Structural and photoluminescence properties of stannate based displaced pyrochlore-type red phosphors: Ca_3−*x*_Sn_3_Nb_2_O_14_:*x*Eu^3+^. Dalton Trans..

[58] Kasturi S, Sivakumar V (2017). Luminescence properties of La_2_W_2−*x*_Mo_*x*_O_9_ (*x* = 0-2):Eu^3+^ materials and their Judd-felt analysis: novel red line emitting phosphors for pcLEDs. Mater. Chem. Front..

[59] Xue J (2017). Improvement of photoluminescence properties of Eu^3+^ doped SrNb_2_O_6_ phosphor by charge compensation. Opt. Mater..

