# Supplementary material for: Eu3+-activated La2MoO6-La2WO6 red-emitting phosphors with ultrabroad excitation band for white light-emitting diodes
Source: Sci Rep. 2017 Sep 20;7:11953. doi: 10.1038/s41598-017-12161-5 (PMC5607311; doi:10.1038/s41598-017-12161-5)
Supplement: Supplementary file 1 — Supplementary Information [file 41598_2017_12161_MOESM1_ESM.doc]

**Supplementary Information**

**Eu^3+^-activated La_2_MoO_6_-La_2_WO_6_ red-emitting phosphors with ultrabroad excitation band for white light-emitting diodes**

**Peng Du and Jae Su Yu***

Department of Electronic Engineering, Kyung Hee University, Yongin-si 446-701, Republic of Korea

***** Corresponding author: jsyu@khu.ac.kr (J. S. Yu)


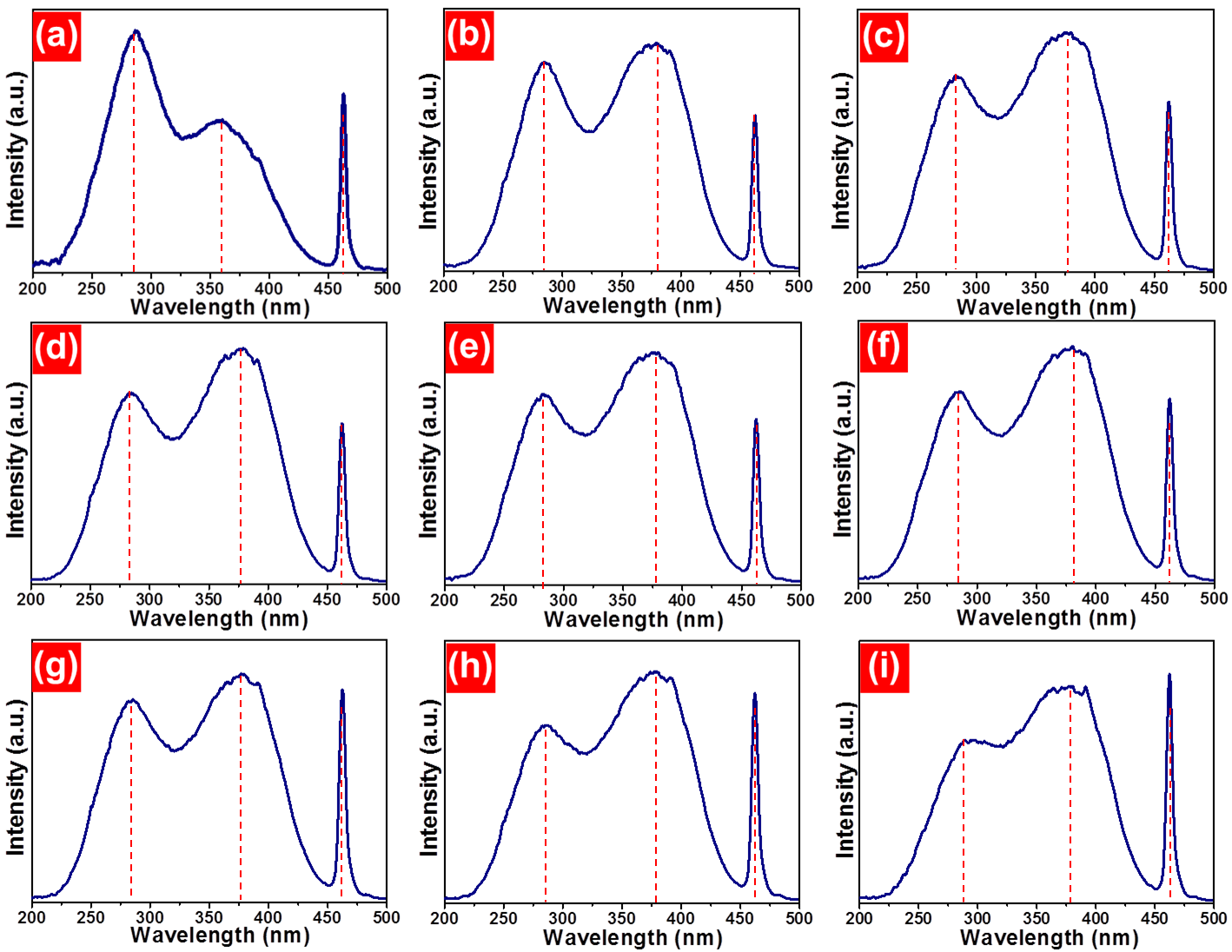


**Figure S1**. Excitation spectra of La_2_MoO_6_-La_2_WO_6_:2*x*Eu^3+^ red-emitting phosphors: (a) *x* = 0.02, (b) *x* = 0.04, (c) *x* = 0.06, (d) *x* = 0.08, (e) *x* = 0.10, (f) *x* = 0.12, (g) *x* = 0.14, (h) *x* = 0.16 and (i) *x* = 0.18.


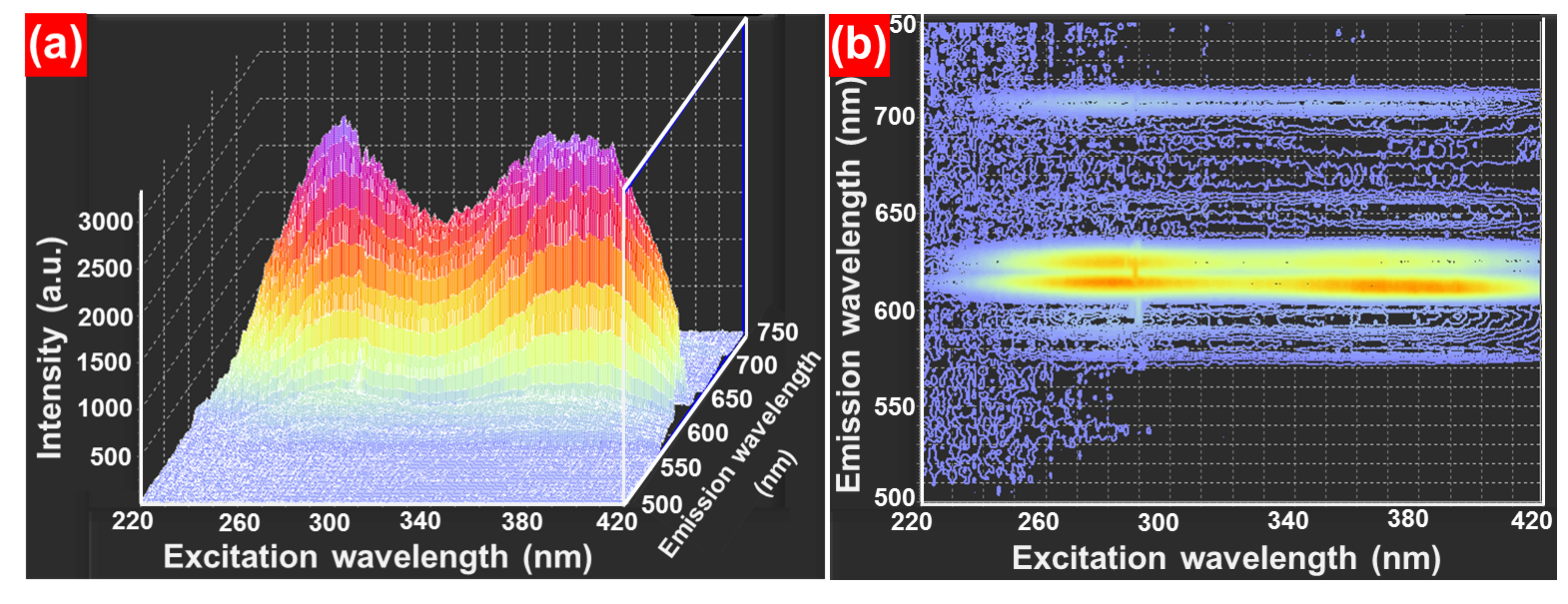


**Figure S2.** (a) 3D emission spectra and (b) contour lines of the La_2_MoO_6_-La_2_WO_6_:0.24Eu^3+^ red-emitting phosphors as a function of excitation wavelength in the range of 220-420 nm.


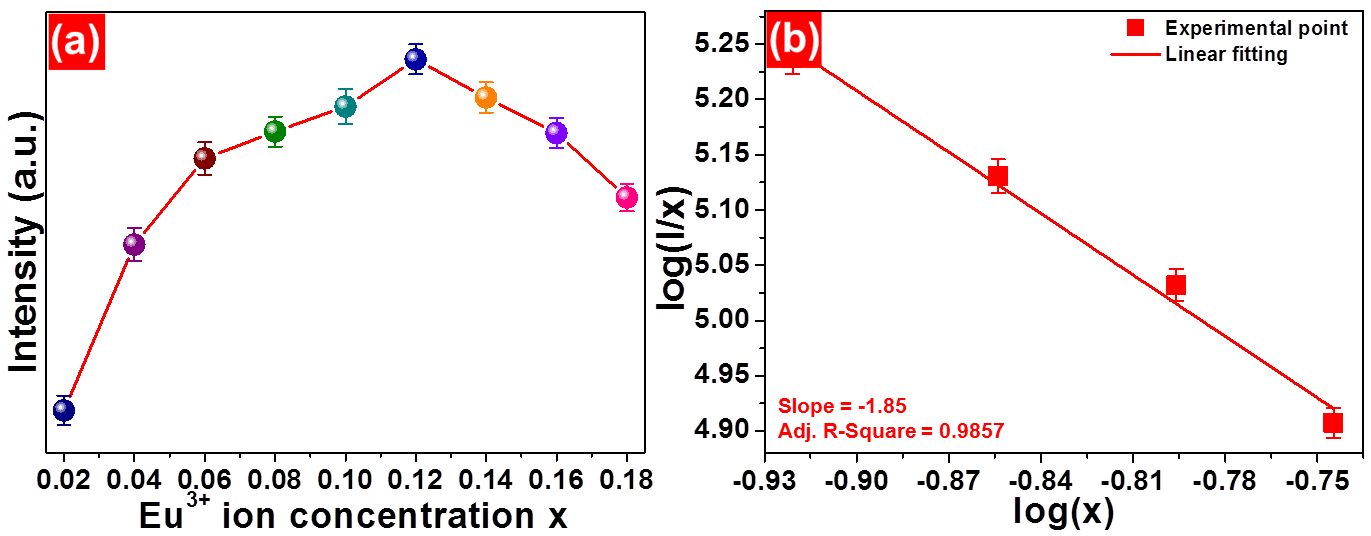


**Figure S3**. (a) Emission intensity as a function of Eu^3+^ ion concentration. (b) Plot of log(*I*/*x*) versus log(*x*).


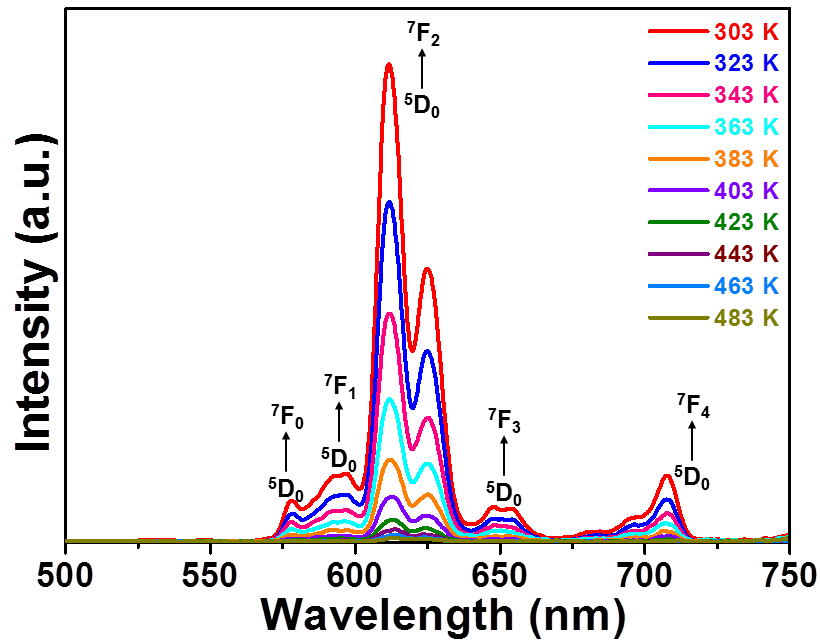


**Figure S4**. Temperature-dependent PL emission spectra of the Eu^3+^-activated LaMoO_6_-LaWO_6_ red-emitting phosphors with the optimal doping concentration.


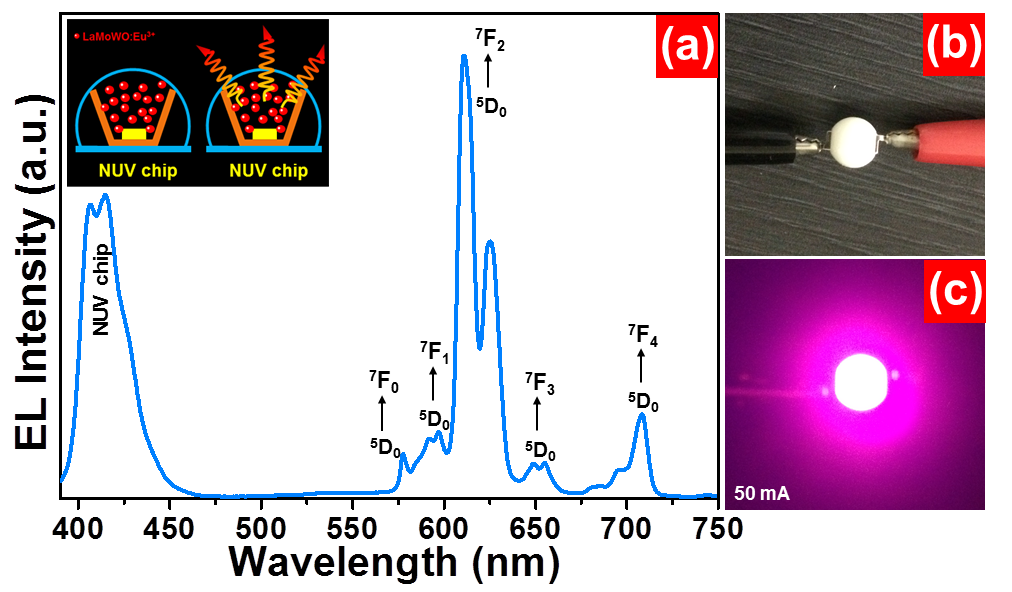


**Figure S5**. (a) EL emission spectrum of the fabricated red-emitting LED device under the forward bias current of 50 mA. (b) and (c) Red-emitting LED device without and with input forward bias current, respectively. Inset shows the schematic diagram of the packaged red-emitting LED device.

**Judd-Ofelt analysis and optical transition parameters**

To get deeper insight into the local structure environment surrounding the Eu^3+^ ions in La_2_MoO_6_-La_2_WO_6_ host lattices, the Judd-Ofelt theory was used to estimate the optical transition parameters, that is, Ω_2_ and Ω_4_. According to Judd and Ofelt’s reports, the probability of the electric dipole (ED) spontaneous emission from the initial *J* state to final *J*´ state can be expressed as: ^1,2^

$A_{ED}=\frac{64\pi^{4}e^{2}}{3h\lambda_{ED}^{3}\left(2J+1\right)}\frac{n\left(n^{2}+2\right)^{2}}{9}\sum_{\lambda=2,4,6} \Omega_{\lambda}\left\langle\psi J\left\|\text{U}^{\lambda}\right\|\psi^{'}J^{'}\right\rangle^{2}$, (1)

where *h* refers to the Plank’s constant, λ*_ED_* is the center wavelength of ED transition, *e* is the elementary charge with a fixed value of 4.8 × 10^-10^ esu, *n* is the refractive index of host (here the value of *n* is about 2.58 deduced from the Gladstone Dale equation) and $\left\langle\psi J\left\|\text{U}^{\lambda}\right\|\psi^{'}J^{'}\right\rangle$ denotes the reduced matrix element for *J* → *J*´ transition. On the other hand, the possibility of magnetic dipole (MD) spontaneous emission from the initial *J* state to final *J*´ state can be calculated by utilizing following expression:

$A_{MD}=\frac{64\pi^{4}n^{3}S_{md}}{3h\lambda_{MD}^{3}\left(2J+1\right)}$, (2)

where λ*_MD_* presents the center wavelength of MD transition. *S_md_* stands for the MD line strength, which is independent of the luminescent host material, and its value is approximately 7.83 × 10^-42^.^3^ As we know, the ^5^D_0_ → ^7^F_1_ transition pertains to the MD transition, while the ^5^D_0_ → ^7^F*_J_* (*J* = 2, 4) transitions are ED transitions.^4^ Therefore, the PL emission intensity ratio (*R*) between the ED and MD transitions can be expressed by

$R=\frac{\int I_{ED}\text{d}\lambda}{\int I_{MD}\text{d}\lambda}$. (3)

According to Equation (1), (2) and (3), the following expression is achieved:

$\frac{\int I_{ED}\text{d}\lambda}{\int I_{MD}\text{d}\lambda}=\frac{A_{ED}}{A_{MD}}=\frac{e^{2}\lambda_{MD}^{3}}{S_{md}\lambda_{ED}^{3}}\frac{\left(n^{2}+2\right)^{2}}{9n^{2}}\sum_{\lambda=2,4,6} \Omega_{\lambda}\left\langle\psi J\left\|\text{U}^{\lambda}\right\|\psi^{'}J^{'}\right\rangle^{2}$. (4)

From the integrated area of emission spectrum, the value of $\int I_{ED}\text{d}\lambda/\int I_{MD}\text{d}\lambda$ can be obtained. Furthermore, the reduced matrix elements are $\left\langle{}^{5}\text{D}_{0}\left\|\text{U}^{2}\right\|{}^{7}\text{F}_{2}\right\rangle^{2}$= 0.0032 and $\left\langle{}^{5}\text{D}_{0}\left\|\text{U}^{2}\right\|{}^{7}\text{F}_{4}\right\rangle^{2}$= 0.0023.^5^ Therefore, by means of Equation (4), the optical transition parameters of Ω_2_ and Ω_4_ for the La_2_MoO_6_-La_2_WO_6_:0.24Eu^3+^ nanophosphors were determined to be about 6.8 × 10^-20^ and 1.2 × 10^-20^ cm^2^, respectively. It is evident that the Ω_2_ is largely dependent on the crystal filed and the large Ω_2_ value means low symmetry of the sites of rare-earth ions, whereas the Ω_4_ is associated with the bulk performance and the rigidity of the luminescent host materials.^6^ Since the calculated Ω_2_ value was much larger than the Ω_4_ value, it is reasonable to consider that the ED transition (^5^D_0_ → ^7^F_2_) takes the domination and the Eu^3+^ ions populate the low symmetry sites in the La_2_MoO_6_-La_2_WO_6_ host lattices, which coincides well with the result obtained from the emission spectra.

**References**

1. Judd, B. R. Optical absorption intensities of rare-earth ions. *Phys. Rev*. **127**, 750-761 (1962).

2. Ofelt, G. S. Intensities of crystal spectra of rare-earth ions. *J. Chem. Phys*. **37**, 511-520 (1962).

3. Wang, L., Guo, W., Tian, Y., Huang, P., Shi, Q. & Cui, C. High luminescent brightness and thermal stability of red emitting Li_3_Ba_2_Y_3_(WO_4_)_8_:Eu^3+^ phosphor. *Ceram. Int*. **42**, 13648-13653 (2016).

4. Tian, Y., Tian, B., Chen, B., Cui, C., Huang, P., Wang, L. & Hua, R. Ionic liquid-assisted hydrothermal synthesis and excitation wavelength-dependent luminescence of YBO_3_:Eu^3+^ nano-/micro-crystals. *J. Alloys Compd*. **590**, 61-67 (2014).

5. Carnall, W. T., Crosswhite, H. & Crosswhite, H. M. Energy level structure and transition probabilities in the spectra of trivalent lanthanides in lanthanum fluoride. *Argonne National Laboratory Report* 1978.

6. Wang, X., Liu, C., Yu, T. & Yan, X. Controlled synthesis, photoluminescence, and the quantum cutting mechanism of Eu^3+^ doped NaYbF_4_ nanotubes. *Phys. Chem, Chem. Phys*. **16**, 13440-13446 (2014).
